# Thoracic Spinal Cord Glioblastoma Mimicking Epidural Abscess: Case Report and Literature Review

**DOI:** 10.7759/cureus.1631

**Published:** 2017-08-31

**Authors:** Rudy Marciano, Zubair Ahammad, Victor Awuor

**Affiliations:** 1 Neurological Surgery, OhioHealth

**Keywords:** glioblastoma, spinal cord, intramedullary, intradural, glioma, epidural abscess, astrocytoma, spinal cord tumor

## Abstract

Spinal cord glioblastoma (SG) accounts for 1.5% of all spinal tumors and has a poor prognosis with survival ranging from 2 to 26 months from presentation.

A 57-year-old male presented with one week of paraparesis and contrasted magnetic resonance imaging (MRI) findings of an epidural enhancing thoracic mass suspicious for an epidural abscess. Intraoperative and pathologic findings revealed SG.

Spinal cord tumors can mimic epidural abscess on MRI. When planning to address extradural spinal pathologies, one should be cognizant of the potential for either isolated or concurrent intradural pathologies. When the epidural findings do not correlate with preoperative imaging, intraoperative ultrasound imaging can identify intradural pathologies without violating the dura.

## Introduction

Spinal cord glioblastoma (SG) is rare, accounting for 1.5% of all spinal tumors [1-2]. The prognosis is poor with survival ranging from 2 to 26 months from presentation [1-2]. Although there is scant data to inform clinical guidelines, the current standard of care, arguably, is maximal safe resection followed by adjuvant chemotherapy and radiation [3-5].

This case report presents a case of thoracic SG closely mimicking an epidural abscess on contrasted magnetic resonance imaging (MRI). The literature on SG treatments and outcomes will be reviewed and discussed. Use of intraoperative ultrasound imaging to identify intradural pathologies will be discussed.

## Case presentation

**Figure 1:**
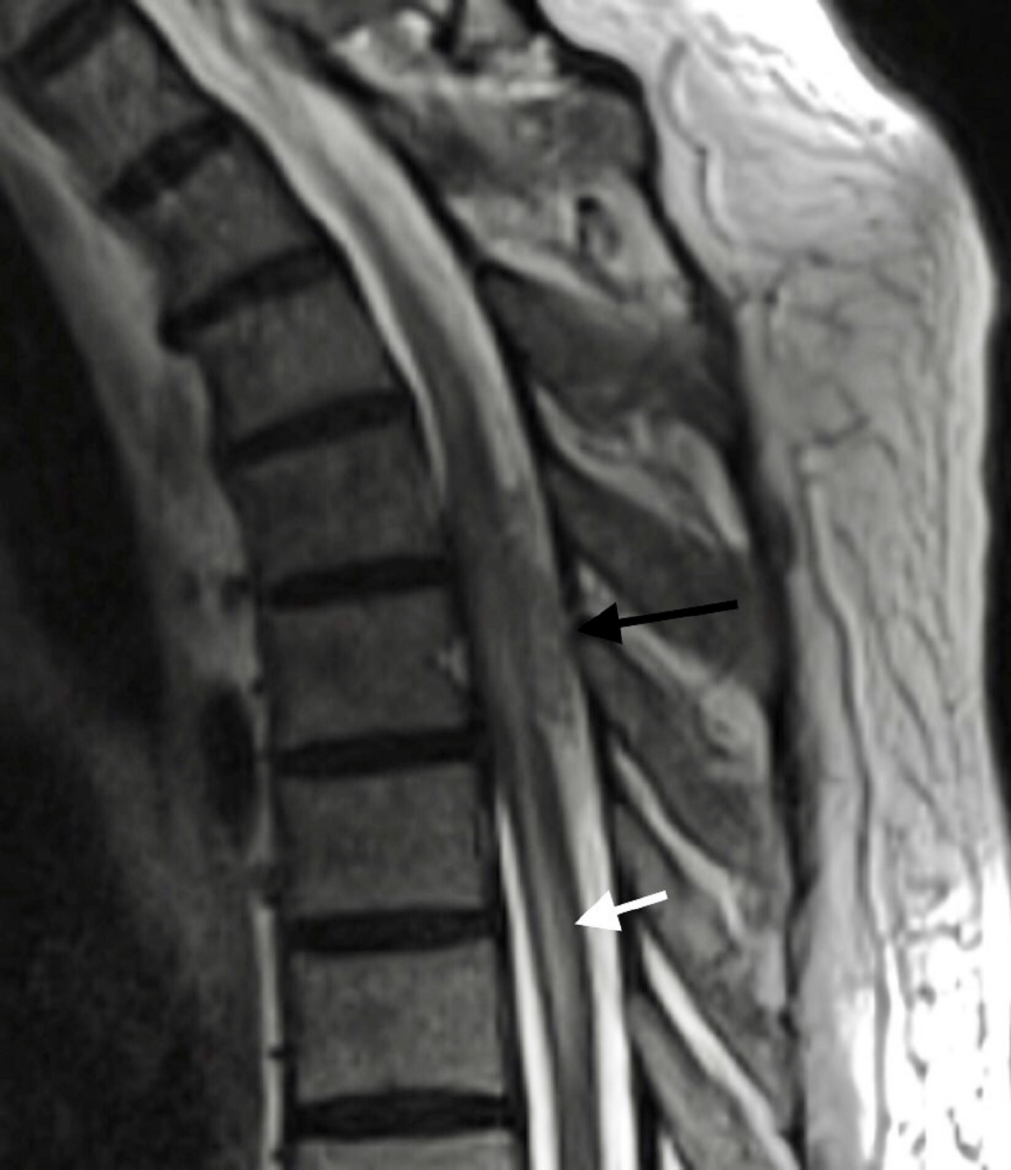
Thoracic T2-weighted magnetic resonance imaging (MRI). Sagittal T2-weighted MRI of thoracic spine at the level of T5 and T6 demonstrating an extraaxial appearing collection (black arrows) and spinal cord edema (white arrow).

**Figure 2:**
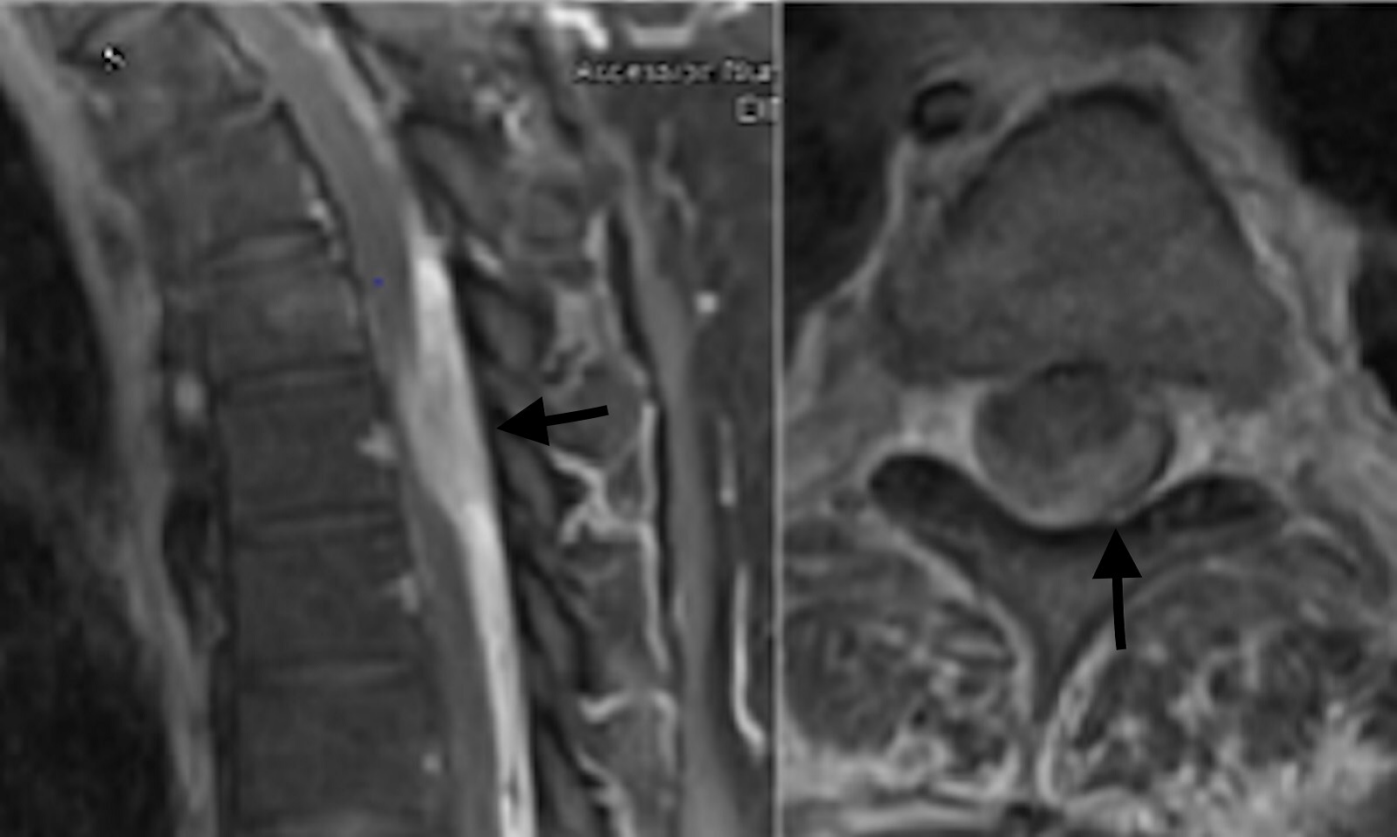
Thoracic T1-weighted magnetic resonance imaging (MRI). Sagittal and axial T1 post-contrast images revealing an enhancing extramedullary appearing lesion (black arrows).

A 57-year-old male patient presented with a one-week history of progressive back pain, paraparesis, and decreased sensation below the umbilicus. His medical and surgical history was unremarkable, including for any previous infectious pathologies or intravenous drug abuse. The patient was afebrile and denied constitutional symptoms. His white blood cell count, erythrocyte sedimentation rate and C-reactive protein levels were not elevated. Physical examination revealed 5/5 bilateral upper extremity strength and 4/5 strength in the lower extremities bilaterally. He had decreased sensation at and below his T10 dermatome. Patellar and Achilles tendon reflexes were 3/4 bilaterally. There were three beats of clonus bilaterally. Muscular tone was normal. Rectal sphincter tone was intact. Enhanced T1 and T2 weighted MRI images of the thoracic spine revealed what appeared to be an enhancing mass within the posterior epidural space at the level of T5 and T6 and extending to the level of the T11 vertebral body (Figures 1, 2).

The patient underwent emergent laminectomies from T4 to T7 but no epidural abscess was appreciated. Intraoperative ultrasound demonstrated an intradural mass. A durotomy along the length of the laminectomy was performed and a large tan intramedullary mass with extramedullary extension was encountered. Subtotal resection was pursued due to unclear gross tumor margins, significant vascularity challenging attempted hemostasis, and the presence of an incomplete preoperative deficit. Frozen section sent intraoperatively was reported as metastatic melanoma versus primary glial tumor. The dura was then primarily repaired and the wound was re-approximated in multiple layers.

**Figure 3:**
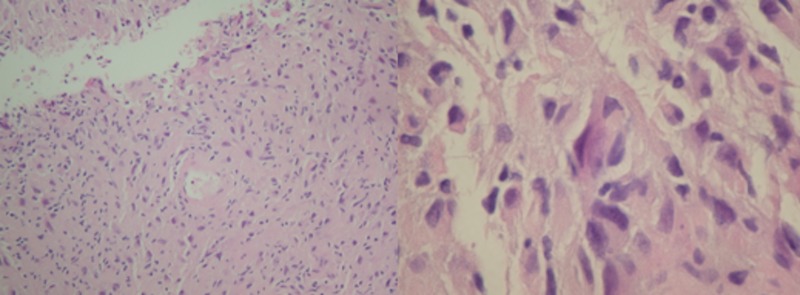
Hematoxylin and eosin stain slides. Hematoxylin and eosin stain at low (left) & high (right) magnification showing epithelioid cells with fibrillar cytoplasm, moderate pleomorphism, occasional nuclear pseudoinclusions and a low mitotic rate.

**Figure 4:**
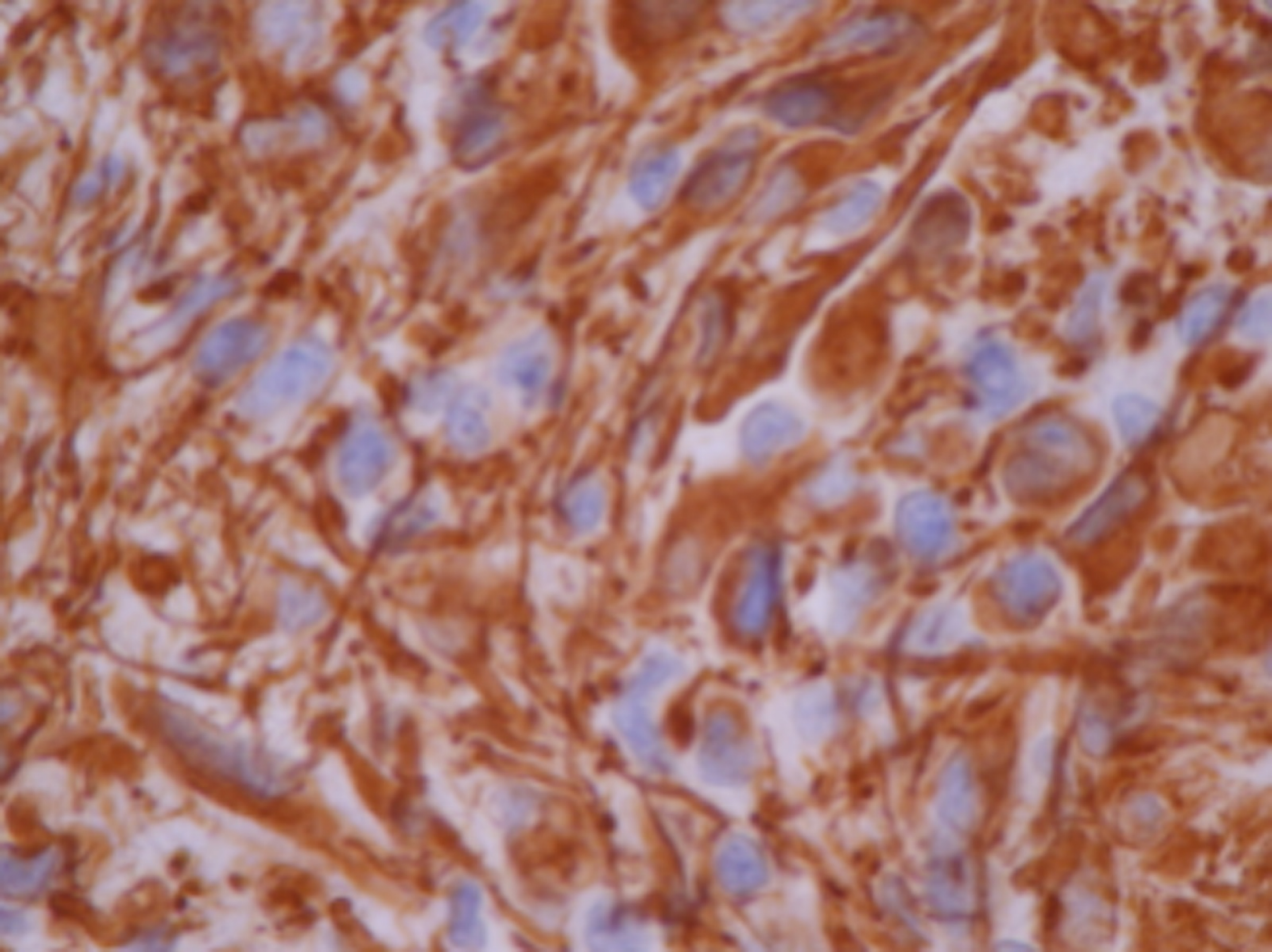
Glial fibrillary acidic protein stain. Glial fibrillary acidic protein stain positivity indicative of astrocytic tumor.

The specimen displayed epithelioid cells with fibrillar cytoplasm, moderate pleomorphism with occasional nuclear pseudoinclusions and a low mitotic rate (Figure 3). There were prominent thick-walled vessels surrounded by an acellular zone as well as zones of acellular fibrillar matrix and scant necrosis. By immunohistochemistry, the tumor cells were positive for GFAP and S-100 protein and negative for CD45, Keratin AE1/3 and melan-A (Figure 4). The final diagnosis was WHO grade IV fibrillary astrocytoma or glioblastoma (GBM).

The patient reported improved sensation in the immediate post-operative period. Lower extremity strength improved to 4+/5 by the time of discharge. Both chemotherapy and radiation therapy were commenced upon discharge. Full neuraxis MRI with and without contrast was negative for concomitant spine or brain lesions.

## Discussion

SG is extremely rare accounting for 1.5% of all spinal tumors and 7.5% of all intramedullary tumors [1-2]. Typical presentation includes the onset of both acute and insidious motor and sensory deficits. On MRI with contrast, SG are characterized by an irregular zone of contrast enhancement. Microscopic features include atypia, mitotic activity, microvascular proliferation, and necrosis [5]. Mean survival grade III and grade IV SG is 12 months [5-6].

Literature guiding management is limited to reviews, case series and reports due to the paucity of this pathology. Confirmed positive prognostic factors including age less than 60, period of therapy occurring after 1980 and adjuvant therapy have been corroborated by multiple series [1,3]. Extent of resection, however, has not been shown to correlate with increased survival and most recent reports indicate subtotal resection or biopsy with or without adjuvant therapy [1,3]. Recommendations for en bloc cordectomy and aggressive resection are sparse but are usually for SG of the conus medullaris with lack of significant neurological function below the tumor [7]. Debulking or biopsy followed by adjuvant focal radiation plus or minus temozolomide chemotherapy appears to be the most common multimodal approach [3-5]. Post-operative radiation with doses in excess of the spinal cord’s tolerance can increase survival to more than 4 years [5,8]. Temozolomide for SG shows only a modest but statistically insignificant survival benefit in retrospective analysis [9]. Certain gene mutations seen in intracranial glioblastoma such as p16, PTEN, BRAF, p53 and H3F3A have been correlated to the development of SG [10]. Future studies will be required to determine if isocitrate dehydrogenase (IDH) mutations or methylation of the O6-methylguanine–DNA methyltransferase (MGMT) gene make adjuvant therapies, such as chemotherapy and radiation, more efficacious in SG as they do in intracranial glioma. Additional potential targets such as hyaluronan and oncolytic viral delivery methods have also been under review [10].

In the presented case, high field strength MRI with contrast did not predict an intramedullary mass. Subtotal resection was completed due to the lack of clear tumor margins, the high level of neurological function in the pre-operative physical exam and the presumed poor prognosis.

## Conclusions

Spinal cord tumors, specifically SG, can mimic epidural pathologies, including epidural abscess, on MRI with contrast. The possibility of a neoplasm should be considered in cases where MRI shows what appears to be an enhancing epidural mass.

Operative planning for extradural spinal lesions should incorporate the remote possibility of intradural pathology. Ultrasound guided localization is valuable in this instance as it allows for intraoperative identification of an intradural lesion without violation of the dura.

Management of SG remains a challenge due to limited research and guidelines for therapy. Surgical debulking and high dose radiation with chemotherapy affords the longest survival to date. Gene therapy holds some promise for the future but further research is necessary to develop optimal treatment strategies.
